# Determinants of microbial contamination of cooked foods hawked in Tharaka Nithi County, Kenya: a cross-sectional study

**DOI:** 10.11604/pamj.2022.41.232.33755

**Published:** 2022-03-22

**Authors:** Cornellius Musembi Muendo, Gideon Kikuvi, Susan Mambo

**Affiliations:** 1 Department of Nursing, School of Nursing and Public Health, Chuka University, Chuka, Kenya,; 2 Department of Environmental Health and Disease Control, School of Public Health, College of Health Sciences, Jomo Kenyatta University of Agriculture and Technology, Nairobi, Kenya

**Keywords:** Determinants, *Escherichia coli*, food hawking, food safety, microbial contamination, *Salmonella*, Shigella

## Abstract

**Introduction:**

hawking of cooked foods is an important socio-economic activity. However, hawked cooked foods are prone to contamination, especially by pathogenic microorganisms.

**Methods:**

this was a cross-sectional study of 151 cooked food hawkers and 151 cooked food samples, one sample each from every respondent. Data was collected through questionnaires, observation checklists, laboratory processes, and key informant interviews. Quantitative data were analyzed using SPSS, while qualitative data were analyzed using the thematic network analysis technique.

**Results:**

adjusted multinomial logistic regression revealed increased odds of microbial contamination in cooked foods hawked by persons with primary-level education (adjusted odds ratio) AOR = 9.278, 95% confidence interval (CI) 2.292, 37.548; p = 0.003), persons earning a profit of Ksh. ≤500.00 (AOR = 4.046, 95% CI 1.825, 8.969; p = 0.001), and in areas infested with rodents (AOR = 6.386, 95% CI 1.877, 21.724; p = 0.003). However, cooked foods hawked by males (AOR = 0.338, 95% CI 0.133, 0.856; p = 0.022), persons trained on food safety (AOR = 0.216, 95% CI 0.064, 0.733; p = 0.014), persons wearing outer garments (AOR = 0.399, 95% CI 0.196, 0.812; p = 0.011), and by persons who stored their garbage in a municipal receptacle or a standard dust bin (AOR = 0.13, 95% CI 0.041, 0.410; p = 0.000) recorded reduced risk of contamination.

**Conclusion:**

hawker´s characteristics, food handling practices, and the condition of the hawking station are important determinants of microbial contamination of cooked foods hawked in Tharaka Nithi County. Targeted interventions need to be developed and implemented to prevent related foodborne diseases.

## Introduction

Hawking of cooked foods is a common worldwide practice. Indeed, some studies have shown that hawking of cooked foods is a valuable cultural and economic activity in many regions of the world [1-5]. The socio-economic benefits notwithstanding, hawking of cooked foods raises serious public health concerns particularly due to the associated poor food handling practices and insanitary food stations. Hawked cooked foods are normally prepared in small factories, hawkers´ homes, or in makeshift structures located in busy streets such as bus parks [6]. A majority of these stations are disorganized, insanitary, dusty, and open to flies, rodents, and insects. Moreover, the stations lack adequate basic sanitation and hygiene facilities such as toilets, hand washing facilities, running water, well-designated utensil washing areas, utensil drying racks, standard solid waste disposal facilities, among others [7,8]. These food safety gaps certainly predispose hawked cooked foods into increased odds of microbial contamination. Food contamination is both a local and a global food safety problem [9-11].

In Kenya, the most common microbial contaminants found in hawked foods include *Escherichia coli, Shigella, Staphylococcus aureus, Salmonella, Clostridium perfringens*, and *Campylobacter jejuni* [7,12]. Thus, this study investigated the risk factors of microbial contamination (with particular focus on *Escherichia coli, Shigella, Staphylococcus aureus*, and *Salmonella*) of cooked foods hawked in Tharaka Nithi County. The specific objectives were determining the association between microbial contamination and hawker´s factors including socio-demographic characteristics and food handling practices, and the association between microbial contamination and hawking site´s environmental conditions. There was barely any empirical information about the determinants of microbial contamination of cooked foods hawked in Tharaka Nithi County (TNC) hence the study provided useful information for targeted food safety interventions.

## Methods

**Study design:** this was a cross-sectional study of 151 cooked food hawkers and 151 hawked cooked food samples.

**Study setting and population:** the study collected data from both the individual hawkers of cooked foods at and their respective hawked cooked foods. A pre-test of data collection tools was carried out at Chogoria Town in October 2019, while the main study was carried out at Chuka Town between January 2021 and April 2022. Chuka Town was purposively selected primarily because it´s the largest and most developed town in Tharaka Nithi County [13]. Chogoria Town compares closely with Chuka Town in key features such as socio-demographic characteristics of her residents, and the climatic and administrative structures. The study respondents included all persons who were displaying for sale a single or more cooked food items during the time of data collection. However, cooked food hawkers below 18 years of age were excluded from participation. In Kenya, persons below 18 years are protected from any activities likely to interfere with their education [14].

**Study variables:** the independent variables were hawker´s socio-demographic characteristics including age, sex, marital status, religion, income earned from hawking cooked foods, main occupation, level of education, and age of business; hawker´s food handling practices including training on food safety, medical examination, possession of food hygiene license, possession of trade permits, availability of handwashing facility, wearing an outer garment, washing hands before handling food, and handling of money. The dependent variable was the status of microbial contamination of hawked cooked foods. A hawked cooked food was considered contaminated when the content of *Escherichia coli* was within the unsatisfactory (>100 cfu/g) category, or when either *Staphylococcus aureus, Salmonella, Shigella*, or more than one of these pathogenic microorganisms were detected.

### Data sources and management

**Data collection tools:** data on the status of microbial contamination of the food samples were obtained through laboratory analysis whereas that on determinants of contamination was collected through questionnaires, observation checklists, and key informants´ interview guides.

**Collection of data from individual respondents:** a route plan was drawn covering all the streets at Chuka Town. Research assistants followed the identified route map collecting data from all available and eligible cooked food hawkers. The eligible respondents were taken through the consent process and requested to voluntarily sign a consent form before data collection.

**Collection of food samples:** all cooked foods from every participating respondent were listed and assigned numerical values based on the order of appearance in the list. A random number generator installed in a mobile smartphone was used to identify a single food item from which about 250 grams were collected. The samples were collected and delivered to the laboratory in their original packaged form, and where possible in whole pieces. All samples were delivered to the laboratory within two hours after collection.

**Detection and enumeration of *Escherichia coli*:** *Escherichia coli (E. coli)* were detected and enumerated based on the food, drugs, and administration procedure [15]. Fifty (50) grams of food sample was blended with 450 ml of butterfield´s phosphate-buffered, from which 5 decimal dilutions were prepared by transferring 1 ml of the original and subsequent mixtures into 9 ml of sterile butterfield´s phosphate-buffered water. Aliquots from each of the dilutions were gently mixed with sterile Violet Red Bile Agar (VRBA) media in separate sterile petri dishes, **allowed to solidify and overlayed with the media**, then incubated at 35°C for 24 hours. All typical colonies were counted and a sample of them was confirmed by incubating in separate tubes of sterile Brilliant Green Lactose Bile (BGLB) broth. Gram staining was carried out to rule out gram-positive, lactose fermenting bacilli, where necessary.

**Detection of *Salmonella*:** twenty-five (25) grams of a representative food sample were successively enriched with sterile nutrient broth and selenite cystine broth. The selectively enriched sample was then streaked on a sterile selective media plate of Xylose Lysine Deoxycholate (XLD) and incubated at 35°C for 24 hours. A portion of typical salmonella colonies were inoculated into sterile Triple Sugar Iron (TSI) slants, and incubated further at 35°C for 24 hours. The presumptive positive results were confirmed by the urease test [16].

**Detection of *Shigella*:** twenty-five (25) grams of a representative food sample were enriched with sterile gram-negative broth and adjusted the pH adjusted to 7 accordingly. The enriched sample was streaked on the surface of sterile MacConkey agar and incubated at 35°C for 24 hours. A portion of typical *Shigella* colonies were inoculated into sterile Triple Sugar Iron (TSI) slants, and further incubated at 35°C for 24 hours. The presumptive positive results were confirmed by the urease test [16].

**Detection of *Staphylococcus aureus*:** twenty-five (25) grams of a food sample were enriched with sterile nutrient broth. The enriched sample was streaked on selective media plates of mannitol salt agar (MSA) and incubated at 35°C for 24 hours. A portion of presumptive *Staphylococcus* colonies were confirmed by coagulase test [16-18].

**Sample size:** since collecting data from the entire estimated population (164) of cooked food hawkers in Chuka Town was feasible, the study carried out a census whereby all (151) cooked food hawkers who met the eligibility criteria and were willing and able to participate got involved. A single sample of cooked food was collected from every study respondent through simple random sampling.

**Data analysis:** quantitative data were analyzed using Statistical Product and Service Solutions (SPSS Statistics 24) while qualitative data were analyzed using the thematic network analysis technique. To identify the determinants of microbial contamination, independent variables were first subjected to a bivariate analysis with the dependent variable using a chi-square test. The independent variables in associations that recorded a p-value of ≤0.3 at the bivariate analysis were subjected to a multivariate analysis using a multinomial logistic regression test [19]. Outputs of multivariate analysis with a p-value of ≤0.05 were considered statistically significant, and the respective independent variables were taken as determinants of microbial contamination.

**Ethical consideration:** this study was reviewed by Chuka University Institutional Ethics Review Committee upon which an ethical clearance reference number CUIERC/NACOSTI/078 was granted. Following the successful ethical review, the study was licensed by the Kenya National Commission for Science, Technology, and Innovation (NACOSTI) through license number 798300. Permission to collect data in Chuka Town was granted by Tharaka Nithi County, letter reference number TNC/CDH/R./VOL1/100. Moreover, the study obtained informed consent from individual respondents before data collection.

## Results

**Socio-demographic characteristics:** the study collected data from 151 hawkers, and 151 cooked food samples, a response rate of 92%. The majority (77.5%) of respondents were females, married (51.7%), and between 35 to 59 years old (71.5%). About half (55.6%) had secondary-level education, while 31.8% and 12.6% had attained primary and tertiary-level education respectively. Almost all (98.7%) of the respondents relied on food hawking for their primary source of livelihood. The hawkers earned a daily net profit of between Ksh 100 to 1,000, with the majority (53.6%) earning a net profit of less than Ksh 500. A bivariate analysis between the status of microbial contamination, and socio-demographic characteristics indicated statistically significant association in levels of education (χ^2^= 13.526, df = 2, p = 0.001) and profit earned from hawking the cooked foods (χ^2^= 9.521, df = 1, p = 0.002). However, when adjusted for sex (p = 0.195), age (p = 0.221) and marital status (p = 0.111) (Table 1), a multivariate logistic regression identified statistically significant associations in sex (p = 0.022), level of education (p = 0.003) and profit earned from hawking cooked foods (p = 0.001). Cooked foods hawked by males (AOR = 0.338, 95% CI 0.133, 0.856) were about three times less likely to be contaminated with microbes compared to those hawked by females. Cooked foods hawked by persons with primary level education (AOR = 9.278, 95% CI 2.292, 37.548) and secondary education (AOR = 4.724, 95% CI 1.351, 16.578) had greater odds of contamination with microbes compared to those hawked by persons with tertiary level education. In terms of profit earned from hawking of cooked foods, the foods hawked by persons earning a profit of Ksh. ≤500.00 (AOR = 4.046, 95% CI 1.825, 8.969) were about four times more likely to be contaminated with microbes compared to those hawked by persons earning between Ksh 501 to 1,000 (Table 2).

**Table 1:** bivariate analysis results for factors associated with microbial contamination of cooked foods hawked in Tharaka Nithi County, 2022

Variable	Contaminated (n/%)	x^2^ Value	df	p-value
**Socio-demographic characteristics, sex (n=151)**				
Male	17/50	1.680	1	0.195
Female	73/62.4			
**Age (in years) (n = 151)**				
18 - 24 years	3/42.9	3.018	2	0.221
25 - 34 years	18/50			
35 - 59 years	69/63.9			
**Marital status (n = 151)**				
Single, never married	30/60	4.402	2	0.111
Currently married	42/53.8			
Separated	90/78.3			
**Education level (n = 151)**				
Primary	36/75	13.526	2	0.001
Secondary	49/58.3			
Tertiary	5/26.3			
**Profit earned from hawking cooked foods (n=151)**				
Ksh. 0 - 500	51/72.9	9.521	1	0.002
Ksh. 501 - 1000	39/48.1			
**Hawker's food handling practices**				
**Trained on food safety (n = 151)**				
Yes	5/33.3	4.773	1	0.029
No	85/62.5			
**Possess a medical certificate (n = 151)**				
Yes	9/56	0.084	1	0.773
No	81/60			
**Possess a food hygiene license (n = 151)**				
Yes	5/45	0.913	1	0.339
No	85/60.7			
**Possess a trade permit (n = 151)**				
Yes	77/62.1	1.792	1	0.181
No	13/48.1			
**Leftover food (n = 151)**				
Yes	84/58.7	0.832	1	0.363
No	6/75			
**Handwashing facility available (n = 151)**				
Yes	18/51.4	1.264	1	0.261
No	72/62.1			
**Designated toilet available (n = 151)**				
Yes	19/65.5	0.521	1	0.470
No	71/58.2			
**Worn outer garment (apron) (n = 151)**				
Yes	30/48.4	5.495	1	0.019
No	60/67.4			
**Washed hands before handling ready-to-eat-food (n = 151)**				
Yes	8/36.4	5.776	1	0.016
No	82/63.6			
**Hawking site's environmental conditions**				
**Food preparation site (n = 151)**				
Home	43/53.6	12.145	2	0.002
Hawking site	42/76.6			
Licensed food premise	5/33.3			
**Means of food transportation (n = 97)**				
Public Vehicle/motorcycle	51/52.6	6.435	1	0.011
Walk (in a bucket or trolley)	46/47.4			
**Source of raw materials (n = 151)**				
Formal business	75/59.5	0.002	1	0.965
Informal business	15/60			
**Storage of garbage (n = 151)**				
Municipal receptacle or standard dust bin	45/46.9	17.732	1	0.000
Indiscriminate dumping or carton or sack	45/81.8			
**Presence of vectors or other pests (n = 151)**				
Yes	60/66.7	4.617	1	0.032
No	30/49.2			

**Table 2:** multinomial logistic regression analysis results for factors associated with microbial contamination of cooked foods hawked in Tharaka Nithi County, 2022

Variable	n/%	COR (95% CI)	AOR (95% CI)	p-value
**Contaminated**				
**Socio-demographic characteristics**				
Sex of respondent				
Male	17/50	0.603 (0.279, 1.301)	0.338 (0.133, 0.856)*	0.022
Female	73/62.4	1	1	
**Education level**				
Primary	36/75	8.4 (2.499, 28.232)*	9.278 (2.292, 37.548)*	0.003
Secondary	49/58.3	3.92 (1.293, 11.888)*	4.724 (1.351, 16.518)*	
Tertiary	5/26.3	1	1	
**Profit earned from food hawking (Ksh.)**				
0 - 500	51/72.9	2.891 (1.495, 5.727)*	4.046 (1.825, 8.969)*	0.001
501 - 1,000	39/48.1	1	1	
**Food handling practices**				
**Trained on food safety**				
Yes	5/33.3	0.3 (0.097, 0.927)*	0.216 (0.064, 0.733)*	0.014
No	85/62.5	1	1	
**Wearing outer garment**				
Yes	30/48.4	0.453 (0.233, 0.883)*	0.399 (0.196, 0.812)*	0.011
No	60/67.4	1	1	
**Hawking site's environmental conditions**				
**Storage of garbage**				
In a municipal receptacle or standard dust bin	45/46.9	0.196 (0.089, 0.434)*	0.13 (0.041, 0.410)*	0.000
In a carton, or sack, or indiscriminate dumping	45/81.8	1	1	
**Presence of rodents at hawking site**				
Yes	60/66.7	2.067 (1.061, 4.024)*	6.386 (1.877, 21.724)*	0.003
No	30/49.2			

*Indicates a statistically significant association (p-value ≤ 0.05) between the category and microbial contamination at a 95% confidence interval

**Food handling practices:** a bivariate analysis between the status of microbial contamination of the food samples and food handling practices indicated statistically significant associations in training on food safety (χ^2^= 4.773, df = 1, p = 0.029), wearing outer garment (χ^2^= 5.495, df = 1, p = 0.019) and washing hands before handling ready-to-eat food (χ^2^= 5.776, df = 1, p = 0.016) (Table 1). However, when adjusted for trade permit (p = 0.181) and handwashing facility (p = 0.261) using multinomial logistic regression, the results indicated statistically significant associations only against training on food safety (p = 0.014) and wearing of outer garments (p = 0.011). Cooked foods hawked by persons trained on food safety were about two times less likely (AOR = 0.216, 95% CI 0.064, 0.733) to be contaminated with microbes as compared to those hawked by persons who had not been trained on food safety. Similarly, cooked foods hawked by persons who had worn outer garments had reduced risk (AOR = 0.399, 95% CI 0.196, 0.812) of microbial contamination compared to those hawked by persons who had not worn outer garments (Table 2). In contrary to the findings of this study, key informants felt that washing hands before handling customers´ food was an important determinant of microbial contamination of hawked cooked foods. However, key informants concurred with the findings of this study that training on food safety and wearing of outer garments were important determinants of microbial contamination of hawked cooked foods.

**Environmental conditions of the hawking sites:** a chi-square test between the status of microbial contamination and the variables related to environmental conditions of the hawking sites revealed statistically significant associations in food preparation site (χ^2^= 12.145, df = 2, p = 0.002), means of transporting the cooked foods (χ^2^= 6.435, df = 1, p = 0.011), storage of garbage (χ^2^= 17.732, df = 1, p = 0.000) and presence of rodents in the hawking site (χ^2^= 4.617, df = 1, p = 0.032) (Table 1). A multivariate analysis of these variables indicated statistically significant associations only in the storage of garbage (p = 0.000) and the presence of vectors or other pests at the hawking site (p = 0.003). The study observed that foods hawked by persons who stored their garbage in a municipal receptacle or a standard dust bin were 0.13 less likely (AOR = 0.13, 95% CI 0.041, 0.410) to be contaminated with microbes of public health importance compared to the foods hawked by persons who stored their garbage in cartons, sacks or just disposed indiscriminately. Poor garbage management practices were also suggested by key informants as a critical determinant of microbial contamination of hawked cooked foods. Cooked foods hawked in areas infested with rodents had 6.386 increased odds (AOR = 6.386, 95% CI 1.877, 21.724) of microbial contamination compared to cooked foods hawked in areas free from rodents and other pests of public health importance (Table 2).

## Discussion

**Socio-demographic characteristics associated with microbial contamination of hawked cooked foods:** sex of the respondent (p = 0.022), level of education (p = 0.003), and profit earned from hawking cooked foods (p = 0.001) were among the determinants of microbial contamination of hawked cooked foods. Cooked foods hawked by males were less likely (AOR = 0.338, 95% CI 0.133, 0.856) to be contaminated with microorganisms of public health importance compared to those hawked by females. Sex was similarly identified as a key determinant of microbial contamination of foods hawked at Bharatpur in Nepal [20]. In Tharaka Nithi, females are socially responsible for the majority of the household chores including taking care of children, management of livestock, and farming, among others. Most of these tasks performed by females at the household level are generally associated with unsanitary processes [21,22] and this could be the reason why cooked foods hawked by females had increased odds of microbial contamination. This is more particularly because the study also showed that the majority of foods were prepared at home. Regarding the level of education, the study showed that cooked foods hawked by persons with primary education and secondary education were about nine times (AOR = 9.278, 95% CI 2.292, 37.548) and four (AOR = 4.724, 95% CI 1.351, 16.578) times respectively more likely to be contaminated with microorganisms of public health importance compared to those hawked by persons with tertiary education.

Similarly, Khadka *et al*. [20] identified level of education as a key determinant of contamination of hawked foods. In Nigeria too, Iwu *et al*. [23] alluded to education as a key determinant of microbial contamination when they found a statistically significant association between knowledge and food hygiene practices. Generally, education improves an individual´s knowledge and skills. In addition, educated persons are increasingly likely to possess good morals and ethical values [24] and this probably explains why the level of education was inversely associated with microbial contamination of hawked cooked foods. Likewise, the profit earned from hawking of cooked foods had a statistically significant negative association with microbial contamination of the hawked cooked foods. Cooked foods hawked by persons who earned a daily profit of Ksh ≤500.00 had about four times (AOR = 4.046, 95% CI 1.825, 8.969) increased odds of microbial contamination compared to the foods hawked by persons earning a daily profit of between Ksh 501 - 1,000. Indeed, a study carried out at Zululand District in South Africa observed that hawkers needed substantial income to meet the recommended food handling requirements [25]. Increased income is known to positively influence household hygiene and especially handwashing and household cleaning [26] and this perhaps explains why cooked foods hawked by persons who earn a daily profit of more than Ksh. Five hundred (500) were less likely to be contaminated as compared to those hawked by persons earning a daily profit of less than Ksh 500. This is particularly plausible since the majority of foods were prepared at home.

**Food handling practices associated with microbial contamination of hawked cooked foods:** in the current study, wearing outer garments (p = 0.011) and training on food safety (p = 0.014) were the only food handling practices that determined microbial contamination of hawked cooked foods. Cooked foods hawked by persons wearing outer garments were almost four times (AOR = 0.399, 95% CI 0.196, 0.812) less likely to contain microbes of public health concern compared to those hawked by persons without outer garments. Kariuki *et al*. [7], Guadu *et al*. [27], and Birgen *et al*. [12] also identified wearing of outer garments as an important determinant of microbial contamination in foods hawked at Nairobi County in Kenya, Gondar Town in Ethiopia, and Nairobi County in Kenya respectively. Outer garments are indicated for wearing by food handlers as protective gear against cross-contamination of ready-to-eat foods from contaminants potentially embedded on the ordinary clothing of the food handlers [28,29]. Consequently, cooked foods hawked by persons without outer garments were found to have an increased risk of contamination with microbes of public health concern. Regarding training, cooked foods hawked by persons who had been trained on food safety were approximately two times less likely (AOR = 0.216, 95% CI 0.064, 0.733) to be contaminated with microbes of public health importance as compared to those hawked by persons who had never been trained on food safety. Similarly, Riyanto *et al*. [30] in a quasi-experimental study carried out in Indonesia to assess the effect of safety education on chemical and bacteriological food safety of foods hawked around public elementary schools at Cimahi City showed that training on food safety was an important determinant of microbial contamination. Training on food safety focuses on building the knowledge and skills of food handlers primarily on the correct measures of enhancing food safety with particular emphasis on prevention of food contamination during preparation, packaging, transport, storage, display, sale to consumption [31,32]. Thus, cooked foods hawked by persons who had not been trained on food safety were found to have increased odds of contamination with microbes of public health importance.

**Hawking site´s environmental conditions associated with microbial contamination of hawked cooked foods:** the environmental conditions that influenced microbial contamination of hawked cooked foods included the method used to store garbage generated from food hawking (p = 0.000) and the presence of rodents and other pests of public health importance at the hawking sites (p = 0.003). Cooked foods hawked by persons who disposed garbage in environmentally friendly ways, particularly in a municipal receptacle or a standard dust bin had reduced risk (AOR = 0.13, 95% CI 0.041, 0.410) of microbial contamination compared to cooked foods hawked by persons who stored their garbage in a carton, sack or just disposed it indiscriminately. Similarly, poor management of garbage generated from food hawking was identified as an important determinant of microbial contamination of foods hawked at Gondar Town in Northwest Ethiopia [27]. Mehboob and Abbas [33] in a study to evaluate the microbial quality of foods hawked at Karachi in Pakistan also alluded that poor waste disposal was potentially responsible for increased microbial contamination of hawked foods. Garbage provides a conducive habitat for rodents and other pests of public health importance such as houseflies and cockroaches [34]. A majority of these pests are known mechanical and biological disease vectors that can depend on hawked foods for breeding, habitat, or for their nutrition [35]. Thus, pests from poorly managed garbage can find their way into nearby human foods and this is perhaps the reason why cooked foods hawked by persons who relied on poor garbage management practices had increased risk of microbial contamination. Moreover, the study showed that cooked foods hawked in stations infested with rodents and other pests of public health importance had about six times (AOR = 6.386, 95% CI 1.877, 21.724) increased risk of microbial contamination compared to foods hawked in areas free from rodents and other pests of public health importance. Similarly, Kariuki *et al*. [7] showed that the presence of rodents and other pests of public health importance was associated with microbial contamination in foods hawked at Nairobi County in Kenya.

**Limitations of the study:** this study analyzed four pathogenic microorganisms to determine the risk factors of microbial contamination of cooked foods hawked in Tharaka Nithi County. Although literature showed that the selected pathogenic microorganisms were the commonly identified contaminants in cooked foods, a focus on more different types of pathogenic microorganisms would have provided a better perspective on the determinants of microbial contamination of hawked cooked foods. This narrow scope on the types of contaminants somehow limited the generalizability of the study findings. The other key limitation was the timing of the study. This study was carried out at a period when the entire world was experiencing a pandemic of COVID-19. In response to this pandemic, the government of Kenya and so Tharaka Nithi County heightened enforcement of COVID-19 control protocols, key among them being the discouragement of non-critical movements of people, especially to crowded places such as towns. This had a ripple effect on the customer base at Chuka Town which somewhat affected the business of hawking cooked foods. The quantities and types of cooked foods hawked at Chuka Town would perhaps be more during normal times. Further, there were accelerated campaigns on handwashing as an important intervention in the prevention of COVID-19. Handwashing, too, is known to significantly reduce microbial contamination of cooked foods. These campaigns may have affected the usual practice of handwashing when handling hawked cooked foods and thus influenced microbial contamination of the analyzed foods.

## Conclusion

The current study has revealed that among the determinants of microbial contamination of hawked cooked foods include sex of respondent, level of education, profit earned from hawking cooked foods, wearing of outer garments, training on food safety, the method used to store garbage and presence of rodents and other pests of public health importance at the hawking stations. County government of Tharaka Nithi and her partners on food safety need to develop targeted policies and programs to prevent potential food-borne infections.

### What is known about this topic

- *Food hawking is a public health concern, particularly due to the associated difficulties in observing food safety standards;*
- *Hawked foods are usually contaminated by diverse environmental contaminants including pathogenic microorganisms, chemicals, and physical agents;*
- *Food hawkers´ characteristics, poor food handling practices, and unsanitary food hawking stations are usually responsible for introduction of microbial contaminants into hawked ready-to-eat foods*.

### What this study adds

- *The study has identified the individual food hawkers´ characteristics, food handling practices, and the specific environmental characteristics of the hawking stations responsible for microbial contamination of cooked foods hawked in Tharaka Nithi County, Kenya;*
- *The study has provided the first-ever empirical evidence on the public health concerns of hawking cooked foods in Tharaka Nithi County, Kenya*.
